# *Leishmania guyanensis* parasites block the activation of the inflammasome by inhibiting maturation of IL-1β

**DOI:** 10.15698/mic2018.03.619

**Published:** 2018-01-14

**Authors:** Mary-Anne Hartley, Remzi O. Eren, Matteo Rossi, Florence Prevel, Patrik Castiglioni, Nathalie Isorce, Chantal Desponds, Lon-Fye Lye, Stephen M. Beverley, Stefan K. Drexler, Nicolas Fasel

**Affiliations:** 1 Department of Biochemistry, University of Lausanne, Epalinges, Switzerland.; 2 Department of Molecular Microbiology, Washington University School of Medicine, St. Louis, Missouri, United States of America.

**Keywords:** Leishmania, Leishmania-virus, metastatic leishmaniasis, inflammasome, NOD-like receptors, RIG-like receptors, A20

## Abstract

The various symptomatic outcomes of cutaneous leishmaniasis relates to the type and potency of its underlying inflammatory responses. Presence of the cytoplasmic *Leishmania *RNA virus-1 (LRV1) within *Leishmania guyanensis*, worsens lesional inflammation and parasite burden, as the viral dsRNA genome acts as a potent innate immunogen stimulating Toll-Like-Receptor-3 (TLR3). Here we investigated other innate pattern recognition receptors capable of reacting to dsRNA and potentially contributing to LRV1-mediated inflammatory pathology. We included the cytoplasmic dsRNA sensors, namely, the RIG-like receptors (RLRs) and the inflammasome-dependent and -independent Nod-like-receptors (NLRs). Our study found no role for RLRs or inflammasome-dependent NLRs in the pathology of *L. guyanensis* infection irrespective of its LRV1-status. Further, neither LRV1-bearing *L. guyanensis* (*LgyLRV1*+) nor LRV1-negative *L. guyanensis *(*LgyLRV1-*) activated the inflammasome *in vitro*. Interestingly, similarly to *L. donovani*, *L. guyanensis* infection induced the up-regulation of the A20 protein, known to be involved in the evasion of inflammasome activation. Moreover, we observed that *LgyLRV1*+ promoted the transcription of inflammasome-independent NLRC2 (also called NOD2) and NLRC5. However, only NLRC2 showed some contribution to LRV1-dependent pathology. These data confirmed that the endosomal TLR3 pathway is the dominant route of LRV1-dependent signalling, thus excluding the cytosolic and inflammasome pathways. We postulate that avoidance of the inflammasome pathways is likely an important mechanism of virulence in *Leishmania* infection irrespective of the LRV1-status.

## INTRODUCTION

The human protozoan parasite *Leishmania* is highly immuno-stimulatory, presenting to the host cell a variety of potent pathogen-associated molecular patterns (PAMPs) that shape the outcome of the inflammatory skin disease known as cutaneous leishmaniasis (CL) 1. CL is a prevalent but neglected tropical disease affecting over 2 million people per year with an expanding geographical reach and a diverse clinical presentation. The various symptomatic outcomes of CL can be categorised by the type and potency of its underlying inflammatory response: ranging from a single, self-healing lesion at the site of inoculation to chronic hyper-inflammatory tissue destruction and the establishment of disfiguring metastatic lesions that are refractory to routine therapies 2. These inflammatory responses have a high-degree of species-specificity, where metastatic and chronic complications occur mainly in infections of the *Leishmania Viannia* subgenus found in Central and South America 3 4.

We previously identified a cytoplasmic dsRNA virus within metastatic *L. guyanensis* (*Lgy*) parasites that acts as a potent innate immunogen capable of worsening lesional inflammation 5 and prolonging infected macrophage survival 6. The dsRNA genome of *Leishmania* RNA virus (LRV1) binds to and stimulates endosomal Toll-Like-Receptor-3 (TLR3), inducing destructive hyper-inflammation 5 and metastasis in an immunosuppressed environment 7. Further, the presence of LRV1 in *L. guyanensis* and *L. braziliensis* negatively affects the patients response to anti-*Leishmania* drugs, as it is predictive of treatment failure and symptomatic relapses 8,9, and potentially worsens the disease outcome in patients co-infected with HIV 10.

By recognising PAMPs and danger-associated molecular patterns (DAMPs) via innate pattern recognition receptors (PRRs), the mammalian immune system can respond rapidly to infection and tissue damage. These PRRs are able to initiate a wide variety of robust inflammatory pathways tailored to suit the stimulatory ligand. Depending on their ligand specificity, localization and signalling pathway, PRRs are classified into three major families: the TLRs (Toll-like receptors), the RLRs (retinoic acid-inducible gene-I-like receptors) 11 and the NLRs (nucleotide-binding-domain, leucine-rich repeat containing receptors).

RLRs are a small family of RNA helicase enzymes found exclusively in the cytosol that launch an anti-viral response after binding cytosolic RNA 12. Most active in dendritic cells (DCs), macrophages and fibroblasts, RLRs comprise of two major family members: RIG-I (retinoic acid-inducible gene-I) and MDA-5 (melanoma differentiation-associated protein 5) that are activated by binding cytosolic RNA. RIG-I typically recognizes 5’ triphosphate short uncapped dsRNA and ssRNA, while MDA-5 recognises longer dsRNA strands 13. Both RIG-I and MDA-5 signal through a common adaptor molecule, MAVS (mitochondrial antiviral signalling protein) to induce a type I interferon (IFN) mediated antiviral response via IRF-3 as well as an NF-(B-type inflammatory response 14. As RLRs are present in the cytosol, their involvement in LRV1-mediated leishmaniasis would indicate that LRV1 is able to exit the phagolysosome, where the parasite survives, and to reach the host cell cytoplasm.

DAMPs such as intracellular content released from dying cells (e.g. nucleic acids, heat-shock proteins and ATP) or damaged components of the extracellular matrix (e.g. hyaluronic acid) are recognised by NLRs. NLR sensing of DAMPs can result in a potent immune response or an inflammatory form of cell death, termed pyroptosis. Additionally, several NLRs are able to respond to PAMPs such as bacterial cell wall components and viral RNA. NLR activation can be accomplished in a fashion similar to that of TLRs, where NF-(B or IRFs are mobilised to produce pro-inflammatory cytokines or type I IFNs, respectively. Alternatively, several NLRs can oligomerise to form a large macromolecular scaffold, called the "inflammasome". Currently, 7 of the 22 human NLR members are known to oligomerise into inflammasomes, namely, NRLB/NLRC4 15 and NLRP-1, -3, -6, -7, and -12 16,17,18,19,20. The inflammasome recruits and activates inflammatory caspases leading to the cleavage and activation of pro-IL-1β and pro-IL-18 21,22. They are formed by the oligomerization of caspase activation and recruitment domains (CARDs), which are either included in the NLR structure such as NLRC4 and NLRP1 or recruited in an ASC (apoptosis-associated speck-like protein containing CARD) protein, which is the case for the formation of the NLRP3, 6, 7, and 12 inflammasome. Additionally, the non-NLR protein, absent in melanoma 2 (AIM2), which contains pyrin and HIN200 domains, senses dsDNA to form an inflammasome by ASC recruitment 23.

To date, only a few studies have thoroughly explored the role of inflammasomes in leishmaniasis. The clearance of *L. amazonensis, L. infantum *and* L. braziliensis *species is correlated to an NLRP3-dependent production of nitric oxide and IFN-γ 24. The authors show that the NLRP3 inflammasome activates IL-1β, and that the IL-1 receptor and its MyD88 adaptor protein are necessary and sufficient to trigger parasitotoxic oxidative stress. Different components of the NLRP3 inflammasome were found to be up-regulated also in a macrophage system of *L. major *infection 25. However, oppositely to the observation in *L. infantum *and *L. braziliensis*, inflammasome activation worsens the outcome of *L. major *infection, accelerating the course of disease or inducing non-healing lesions 26,27. Predictably, the effects of inflammasome activity could differ widely amongst various *Leishmania* species, ranging from protective in* L. amazonensis, L. infantum *and* L. braziliensis* infection 24 to pathogenic in cutaneous species from the Paleotropics 26,27 and contrasting in visceralizing *L. donovani*, where it has been involved in the development of protective immunity 28 but has also recently been correlated with increased visceralization if activated by gut bacteria transmitted during a sand fly bite 29. Of note, however, IL-1α, IL-1β and IL-18 can be activated independently of the inflammasome 30 and thus these roles are not necessarily indicative of NLR activity. Recently, A20 (also named TNF-α-induced protein 3), a deubiquitinating protein, was reported to inhibit NF-κB activation by deubiquitinating TRAF6 and also to block inflammasome activation by inhibiting IL-1β maturation independently of its effect on NF-kB 31. In *L. donovani* infection, A20 was shown to inhibit TLR signalling, decreasing the pro-inflammatory response, thus favouring parasite survival 32 by blocking both pro-IL-1β and pro-caspase-1 maturation 33.

In this study, we investigated the role of several non-TLR3 pathways of viral dsRNA recognition during LRV1-mediated pathology in a murine model of *Lgy* infection. To this end, we performed an expression array of NLR and RLR components in *Lgy* infection, probing for markers of *Lgy* induced inflammasome activation at a cellular level. We then tested the role of these components on LRV1-dependent leishmanial pathology in a murine model of infection.

## RESULTS 

### LRV1 does not activate RLR signaling

**Figure 1:**
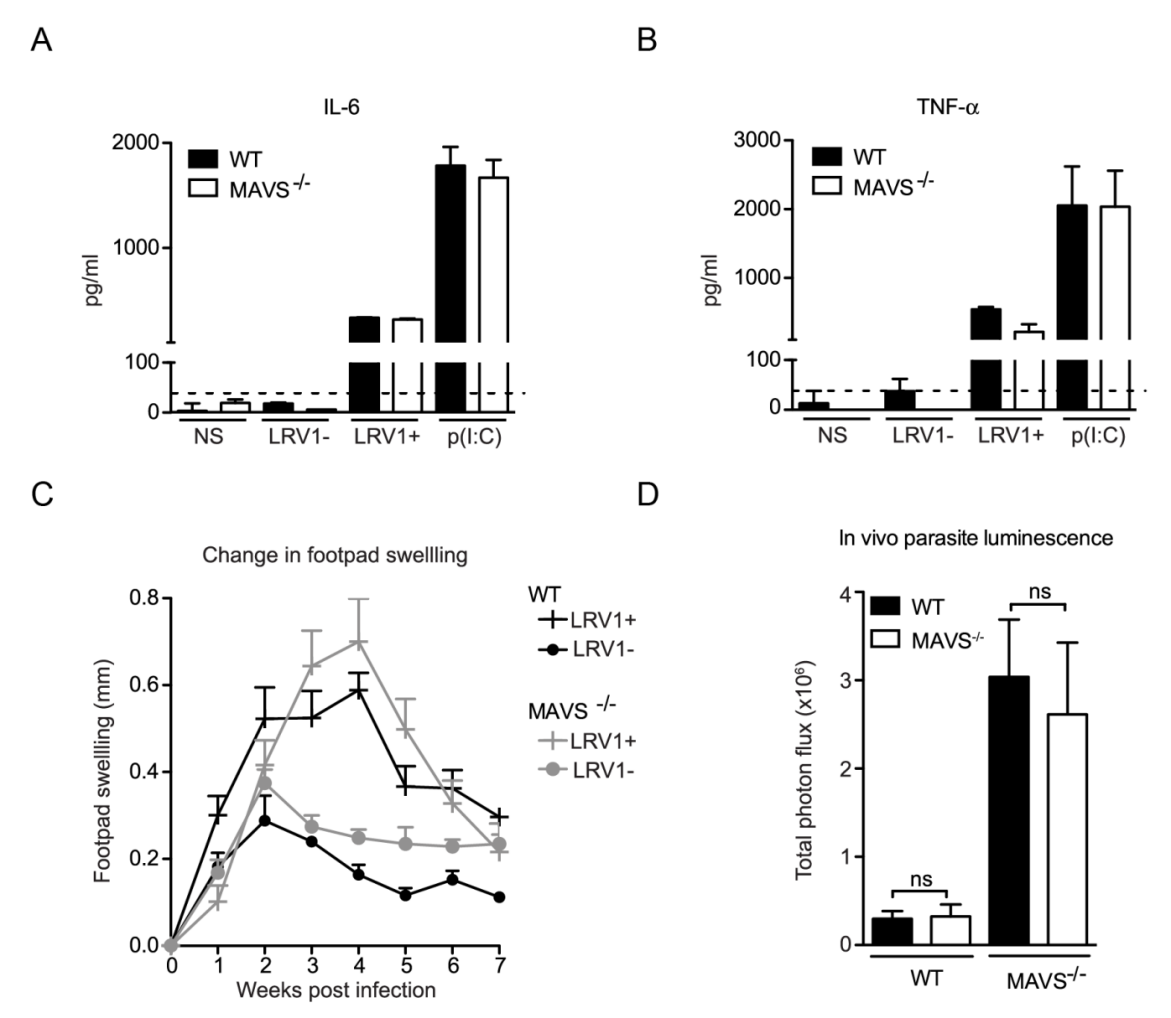
FIGURE 1: LRV1 does not activate RLR signaling. **(A, B)** BMDMs from mice deficient in MAVS and their C57BL/6 WT counterparts were infected with *LgyLRV1+ *or *LgyLRV1-* parasites or stimulated with a synthetic TLR3 ligand, poly(I:C). After 24 hours, the NF-(B cytokines, IL-6 **(A)** and TNF-α **(B)** in BMDM supernatant were quantified by ELISA. **(C, D)** MAVS^-/-^ and C57BL/6 WT mice were infected with* LgyLRV1+ *or *LgyLRV1-* parasites in their hind footpads. **(C)** Weekly lesional swelling was monitored as a proxy for disease progression. **(D)** At the peak of infection (4 weeks), parasite burden was measured by *in vivo* luminescence, by injecting mice intra-peritoneally with 15 mg/kg luciferin. Data is representative of a minimum of three independent experiments, using at least five mice per condition, and showed as mean ± SEM. Significance tested by Student’s* t-test* (bar graphs) or *one-way Anova* (disease score), ns: non-significant.

MAVS is the common adaptor molecule essential in the signalling of RIG-I and MDA-5 34. Therefore, to investigate whether RLRs contributed to LRV1-mediated parasite survival and inflammation, we infected bone marrow derived macrophages (BMDMs) from C57BL/6 (WT) and MAVS^-/-^ mice with *LgyLRV1+* or *LgyLRV1-* parasites. We found that the absence of MAVS did not affect LRV1-induced up-regulation of the signature NF-(B cytokines IL-6 and TNF-α (Fig 1A and 1B). Subsequently, to include the effect of all cells in which RLRs may be activated in the context of the leishmanial lesion, we monitored disease progression in a murine model of infection. Here, mice lacking MAVS signalling did not show any visible variation in lesional swelling (Fig 1C) or parasite burden (Fig 1D), as compared to the WT control. Thus, we could exclude RLRs as sensor molecules participating in an LRV1-dependent exacerbation of the disease and conclude that LRV1 dsRNA does not reach the host cell cytoplasm.

### LRV1 promotes the transcription of the inflammasome components, caspase-1, IL-1β, NLRP3 and AIM2

**Figure 2:**
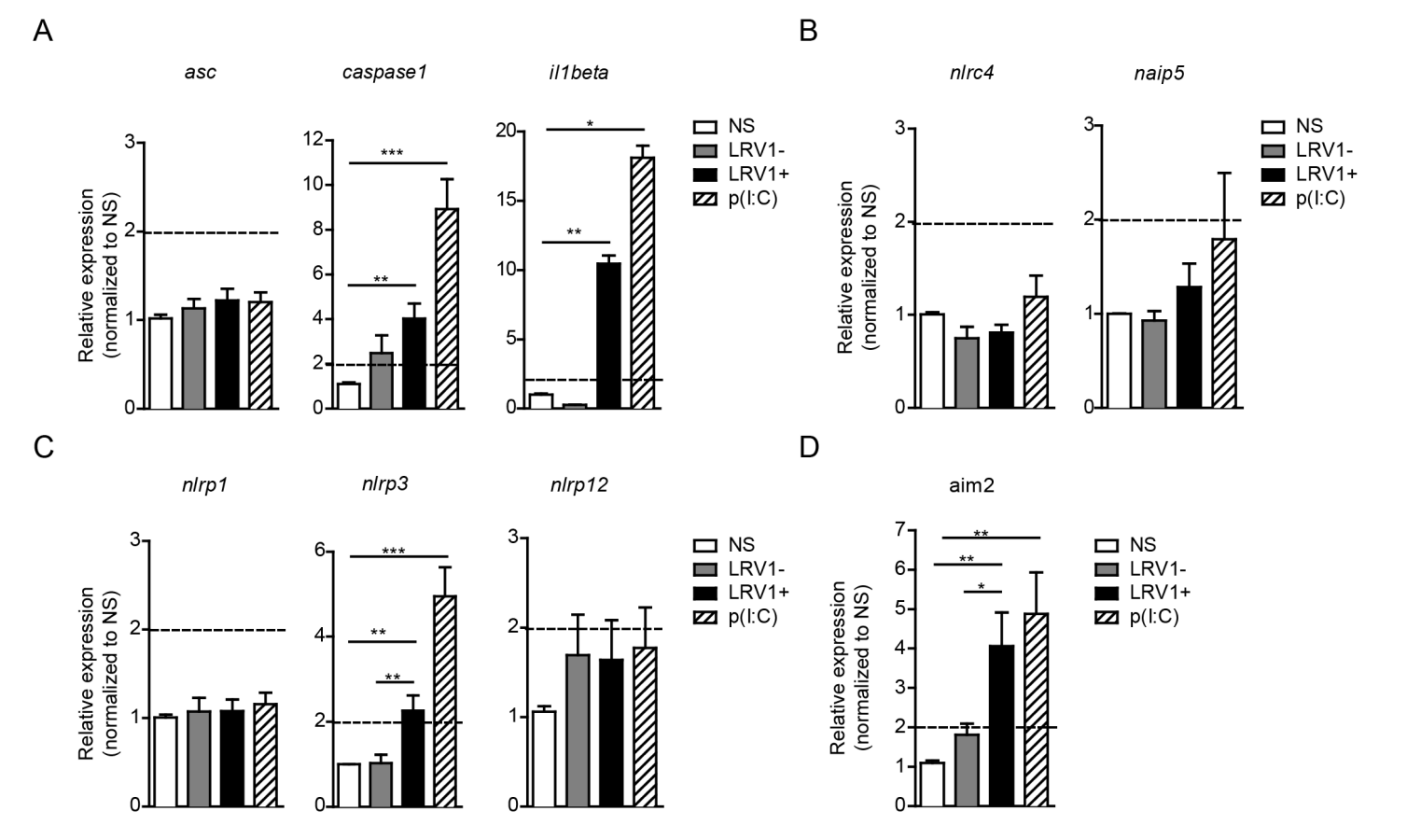
FIGURE 2: LRV1 promotes the transcription of the inflammasome components, caspase-1, IL-1β, NLRP3 and AIM2. BMDMs from C57BL/6 mice were infected with *LgyLRV1+ *or *LgyLRV1-* parasites or stimulated with a synthetic TLR3 ligand, poly(I:C). After 6 hours and 12 hours, mRNA was quantified by qRT-PCR. **(A)** The common inflammasome components ASC, Caspase-1 and IL-1β and the inflammasome-forming members of the **(B)** NLRC family, **(C)** NLRP family and **(D)** a non-NLR inflammasome-forming molecule, AIM2 were measured. Levels are representative of both 6-hour and 12-hour time points. Representative results are shown from at least three independent experiments, using two technical and two biological replicates, and showed as mean ± SEM. Non-parametric Student’s* t-test* is shown. *p < 0.05, **p < 0.01, ***p < 0.001. A cut off value of 2-fold was used as a limit of significance and is indicated as a dotted line.

To identify the possible interaction between LRV1 and the inflammasome, we performed an expression assay of the key inflammasome signalling transducers as well as the major inflammasome-forming PRR family. We focused on genes encoding the common inflammasome components *asc*, *caspase 1* and *il1beta* (Fig 2A), and the inflammasome-forming members *nlrc4* and *naip5 *(Fig 2B)*, *the NLRP family members *nlrp1*, *nlrp3*, *nlrp12 *(Fig 2C) and a non-NLR inflammasome-forming molecule* aim2 *(Fig 2D)*.* BMDMs were infected with *LgyLRV1+ *or *LgyLRV1-* parasites, or stimulated with the synthetic TLR3 agonist, poly(I:C), and then prepared for qRT-PCR analysis. We found that TLR3 stimulation was able to potently induce the transcription of genes including *caspase-1*, *IL-1*β, *nlrp3* and *aim2 *but not *asc*, *nlrc4*, *naip5*, *nlrp1* and *nlrp12 *(Fig 2). Taken together, these results showed that LRV1 and poly(I:C) mediated TLR3 activation can prime the inflammasome components and promote the transcription of certain NLRs and AIM2.

### L. guyanensis does not activate the inflammasome in macrophages, irrespective of its LRV1 status

Based on the transcriptional data, we could expect that LRV1 would activate the inflammasome. Such activation involves the autocatalytic cleavage of pro-caspase 1 into its p20 and p10 subunits. In turn, active caspase-1 cleaves and activates pro-IL-1β and pro-IL-18. Importantly, while pro-IL-18 and pro-caspase-1 are constitutively expressed, the transcription of pro-IL-1β is under the control of NF-κB. Therefore, inflammasome activity requires a "priming" step via NF-κB activation before a second signal activates the inflammasome. NF-κB-dependent TLR3 signalling as observed in dsRNA activation is one such pathway leading to inflammasome priming 22,35.

**Figure 3:**
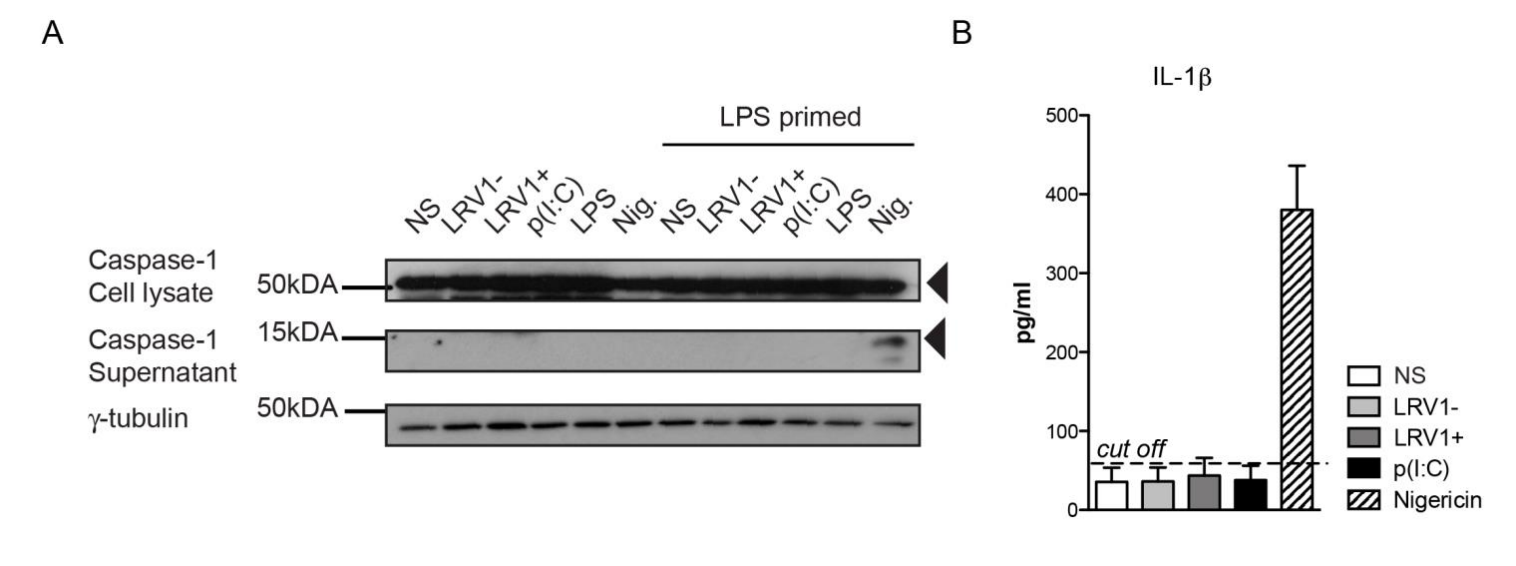
FIGURE 3: *L. guyanensis* does not activate the inflammasome in macrophages, irrespective of its LRV1 status. **(A-B)** BMDMs from C57BL/6 WT mice were primed with 200 ng/ml of LPS for 3 hours. Subsequently, cells were incubated with *LgyLRV1+ *or *LgyLRV1-* parasites, poly(I:C) or culture medium. Additionally, a 1 hour treatment with 5 µM of Nigericin was used as a positive control for the inflammasome activation. Proteins were extracted from cell lysates (CL) and supernatants (SN). **(A)** Cell lysates were blotted for caspase-1. **(B)** IL-1β detected by ELISA in cell-free supernatant. Graphs are representative of a minimum of three independent experiments, using four technical and two biological replicates, and showed as mean ± SEM. Non-parametric Student’s* t-test* was used to calculate significance.

To investigate whether we were able to activate the inflammasome in our BMDMs, we primed BMDMs with lipopolysaccharide (LPS) for 3 hours before *LgyLRV1+* or *LgyLRV1-* infection, and added nigericin treatment as a positive control of inflammasome activation. Cell lysates and cell culture supernatants were probed for the pro- and cleaved forms of caspase-1 by immunoblotting (Fig 3A). *LgyLRV1+* infection or poly(I:C) treatment increased the expression of the pro-form of IL-1β, and thus acted as priming signals. However, *L. guyanensis *parasites, irrespective of their LRV1 status did not activate the inflammasome in BMDMs, since the IL-1β cytokine and cleaved active caspase-1 could not be detected in the cell supernatant (Fig 3A and 3B). Taken together, we were able to definitively exclude inflammasome activation in the macrophage host cell during infection with *L. guyanensis* parasites.

#### The inflammasome does not contribute to disease pathology in a murine model of Lgy infection, irrespective of its LRV1 status

**Figure 4:**
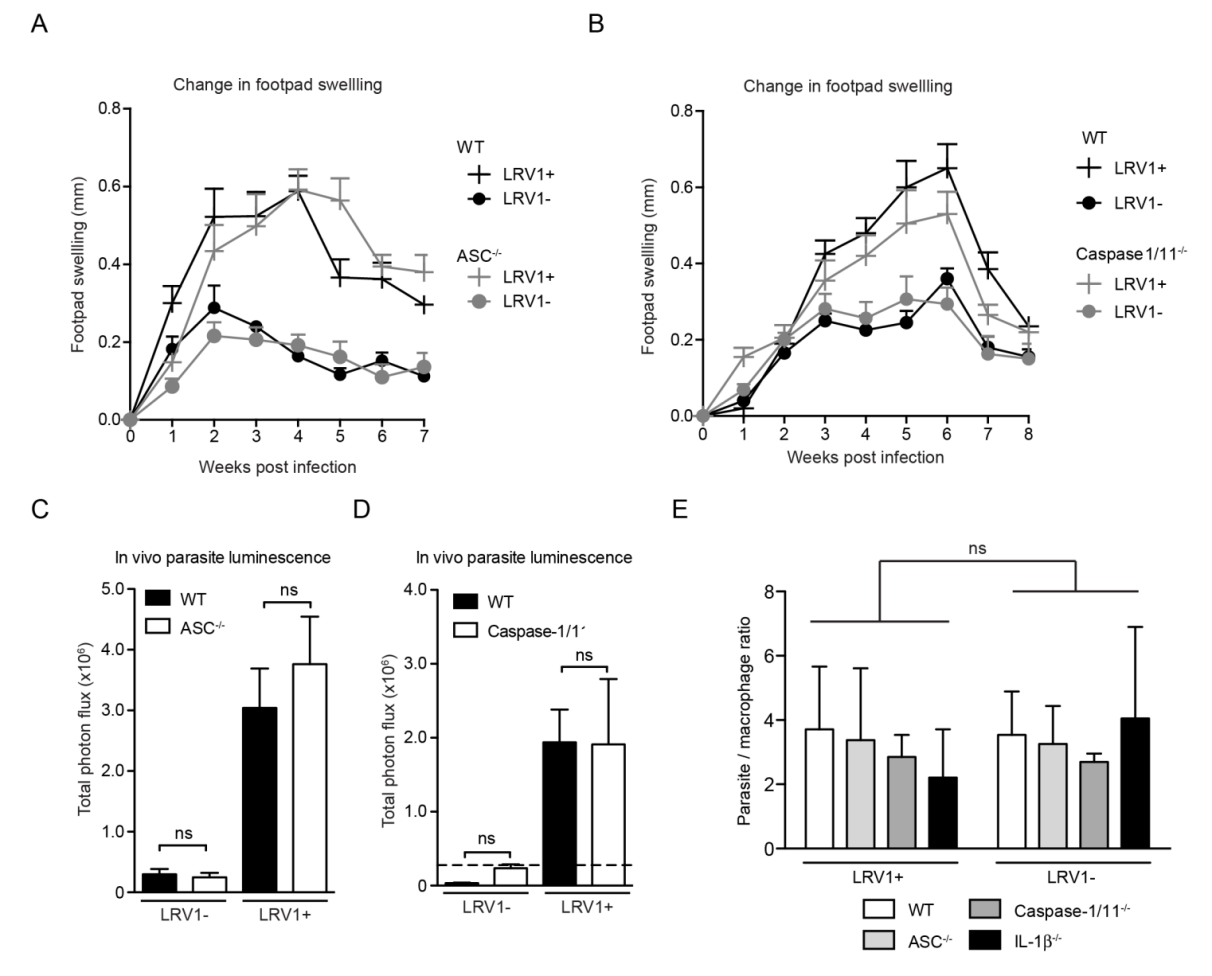
FIGURE 4: The inflammasome does not contribute to disease pathology in a murine model of Lgy infection, irrespective of its LRV1 status. WT, ASC-/- and Caspase1/11-/- mice were infected with LgyLRV1+ or LgyLRV1- parasites in their hind footpads. **(A, B)** Weekly lesional swelling was monitored as a proxy for disease progression. **(C, D)** At the peak of infection (4 weeks), parasite burden was measured by *in vivo* luminescence, by injecting mice intra-peritoneally with 15 mg/kg luciferin. **(E)** BMDMs derived from C57BL/6 mice deficient in ASC, Caspase-1/11 and IL-1β were infected with either *LgyLRV1+* or *LgyLRV1-* parasites for 24 hours then stained with DAPI and acquired using high-content microscopy. Parasite burden was unbiasedly quantified using computational image analysis. The data shown is representative of a minimum of three independent experiments, using at least 5 mice per condition, and showed as mean ± SEM. Statistical significance was quantified by performing Student’s t-test (bar graphs) or *one-way Anova* (disease score), ns: non-significant.

As the macrophage is just one of the many cells with potential inflammasome activity in the skin 36,37, it was important to verify inflammasome activity in the physiological context of an *in vivo* infection. Using mice deficient in caspase1/11 (thus lacking both the canonical caspase-1-dependent and non-canonical caspase-11-dependent pathway of inflammasome activation 38) or ASC, we found no significant differences in the development of lesional swellings for *Lgy* infection, irrespective of LRV1 presence (Fig 4A and 4B). These mice also did not display any differences in parasite burden at the peak of infection as quantified by *in vivo* luminescence (Fig 4C and 4D)**. **Moreover, the conditional knockouts of a floxed *asc* gene using CRE expression in DCs, macrophages or keratinocytes mediated by promoters *Itgax* (CD11c), *lysosome 2 *(LysM) and *keratin 14* (K14), respectively, did not affect the LRV-mediated disease progression in mice, thus allowing us to exclude the contribution of the inflammasome of other cell types such as DCs, neutrophils and keratinocytes that are present in lesions (Fig S1). Consistent with these results, the parasite burden in macrophages deficient in ASC, caspase-1 and IL-1β were similar to WT macrophages 24 hours post infection (Fig 4E). In conclusion, the inflammasome did not have any effect on lesional pathology or parasite burden in *Lgy* infection irrespective of its LRV1 status.

#### *L. guyanensis* induces the expression of the inflammasome-inhibitor protein A20

**Figure 5:**
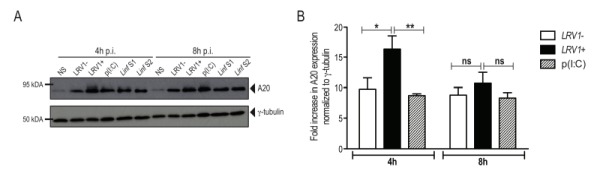
FIGURE 5: : *L. guyanensis* and *L. infantum *infection induce the up-regulation of the inflammasome inhibitory factor, A20. BMDMs from C57BL/6 WT mice were infected with *LgyLRV1+, LgyLRV1-*, *Linf *S1 or *Linf* S2 parasites or stimulated with poly(I:C) (p(I:C)) as a positive control for TLR3 activation. **(A)** At 4 or 8 hours after infection, cells were collected, proteins were extracted from cell lysates and were blotted for A20 and γ-tubulin. **(B)** Quantification of protein expression of *LgyLRV1+ *or *LgyLRV1- *infected or poly(I:C) treated macrophages. The graph is representative of four independent experiments, and shown as mean ± SD. *Two-way ANOVA* was used to calculate significance. *p < 0.05; **p < 0.01; ns: non-significant.

Recently, A20 was further shown to promote *L. donovani* virulence, as it inhibits inflammasome formation possibly by blocking pro-IL-1β and procaspase-1 maturation 33. Considering that we observed an inhibition of the activation of the inflammasome, we investigated whether *LgyLRV1+* or *LgyLRV1-* could induce the expression of A20. We therefore infected BMDMs with *LgyLRV1+* or *LgyLRV1- *and analysed cell lysates for increased expression of A20 by immunoblot at 4 and 8 hours post infection. As a positive control, we infected BMDMs with two strains of *L. infantum* and analysed A20 expression. Similarly to *L. infantum*, in our model of macrophage infection, *LgyLRV1+* and *LgyLRV1-* induced the expression of A20 (Fig 5A), suggesting its possible involvement in the inhibition of inflammasome activation by blocking IL-1β maturation 31. To better assess LRV1 involvement in A20 induction, we quantified by densitometric analysis the expression of A20 in *LgyLRV1+ *or *LgyLRV1-* infected or poly(I:C) treated macrophages. Interestingly, at early time points, infection with *LgyLRV1+* parasites significantly increased A20 expression compared to both *LgyLRV1-* infection or poly(I:C) treatment, suggesting an additive effect between *Leishmania* infection and TLR3 stimulation (Fig 5B).

### LRV1 induces the expression of inflammasome-independent NLRs: NLRC1, NLRC2 and NLRC5

Many of the inflammasome-independent NLR family members are able to recognise viral components and often use signalling pathways similar to that of the TLR3-LRV1 response. To identify NLRs of interest in our model, we screened BMDMs for LRV1-induced expression of the inflammasome-independent NLR members that are known components of the anti-viral response.

**Figure 6:**
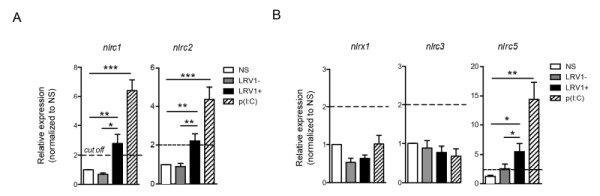
FIGURE 6: LRV1 induces the expression of inflammasome-independent NLRs: NLRC2 and NLRC5. **(A-B)** BMDMs from C57BL/6 mice were infected with *LgyLRV1+ *or *LgyLRV1-* parasites or stimulated with a synthetic TLR3 ligand, poly(I:C). After 6 hours, mRNA was quantified by qRT-PCR for NLRC1 and NLRC2 **(A)** or for NLRC5, NLRX1 and NLRC3 **(B)**. The bar graphs are representative of at least 3 independent experiments, using 2 technical and 2 biological replicates, and showed as mean ± SEM. Non-parametric Student’s *t*-test was performed. *p < 0.05, **p < 0.01, ***p < 0.001. A cut off value of 2-fold was shown as a dotted line.

NLRC2 can act as a cytoplasmic PRR for ssRNA, the replicative RNA and mRNA of *Totiviridae* 39, and signal through MAVS to promote IFN-β transcription via IRF3 40. However, in macrophages stimulated with poly(I:C) NLRC2 is not implicated in the production of IFN-β, TNF-α and IL-6 40. Oppositely, it was reported that NLRC1 and NLRC2 are induced in response to viral infection, poly(I:C) or IFN-β stimulation and to enhance the production of pro-inflammatory cytokines 41. Further, NLRC2 promotes the CCL2-dependent recruitment of inflammatory monocytes 42. To test for the expression of NLRC1 and NLRC2 in our *in vitro *infection model, we infected WT BMDMs with *LgyLRV1+* or *LgyLRV1-* parasites, or stimulate them with poly(I:C). Six hours post infection, BMDMs were lysed and the expression of different NLR transcript was measured by qRT-PCR. In accordance with published data, we observed that both LRV1 and poly(I:C) stimulation promoted the transcription of *nlrc1* and *nlrc2* (Fig 6A). Further, it was reported that NLRX1 43, NLRC3 44 and NLRC5 45,46 negatively regulate mitochondrial antiviral immune signalling. Here, we observed that the stimulation of macrophages with poly(I:C) or *LgyLRV1+* resulted in the induction of *nlrc5* (Fig 6B), whereas the expression of *nlrx1* and *nlrc3* was unaltered (Fig 6B). Together, these results indicated that LRV1-mediated TLR3 activation can act as a priming signal for certain inflammasome-independent NLRs.

### NLRC2 deficiency in mice reduces LRV-mediated pathogenesis 

**Figure 7:**
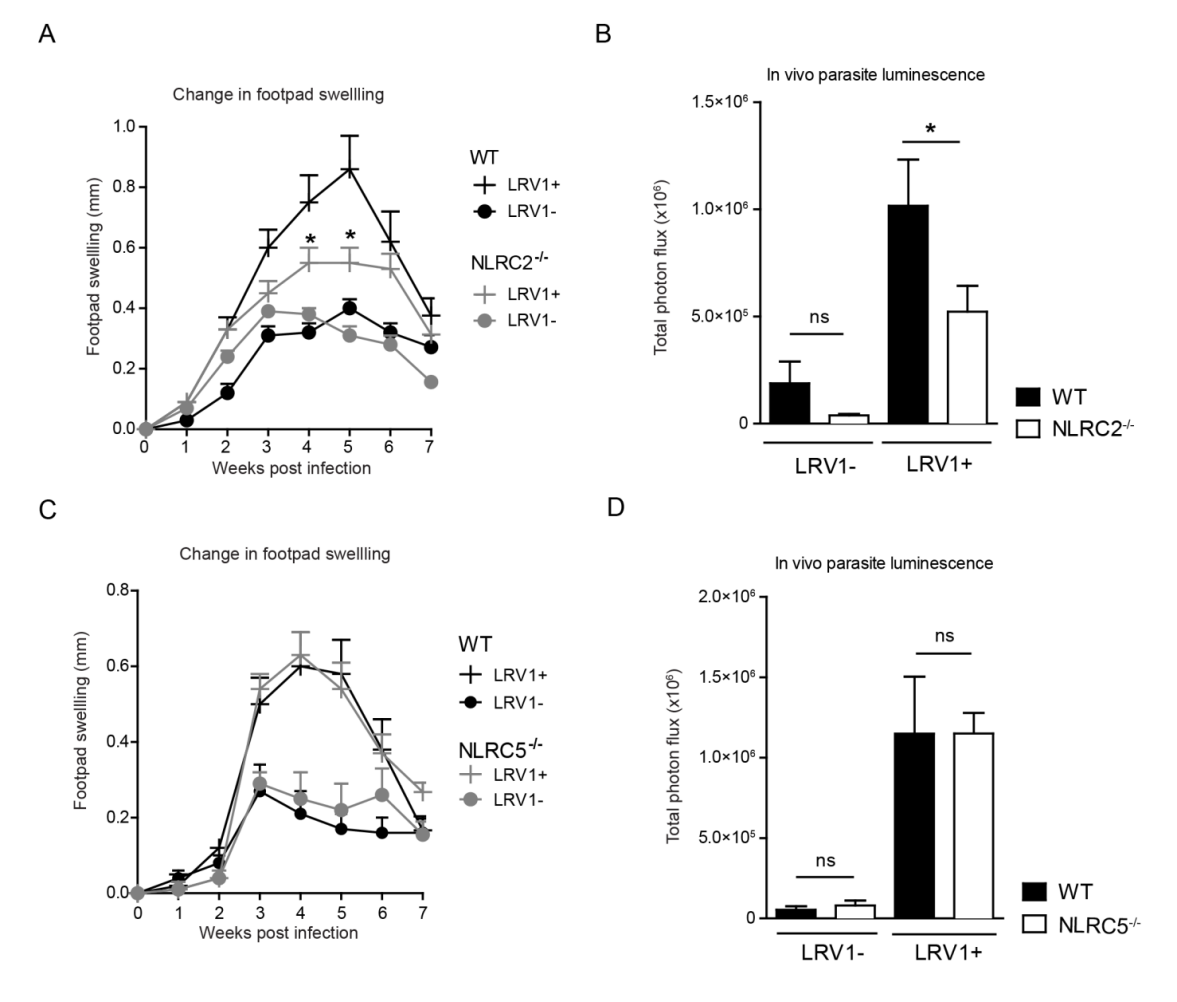
FIGURE 7: NLRC2 reduces LRV-mediated pathogenesis. **(A-D)** Mice were infected with *LgyLRV1+* or *LgyLRV1-* parasites in their hind footpad. Weekly lesional swelling in infected NLRC2^-/- ^**(A)** and NLRC5^-/- ^**(C)** was monitored as a proxy for disease progression. At the peak of infection (4 weeks), parasite burden in infected NLRC2^-/- ^**(B)** and NLRC5^-/- ^**(D)** mice was measured by *in vivo* luminescence, by injecting mice intra-peritoneally with 15mg/kg luciferin. The representative graph is shown from at least three independent experiments, using two biological replicates in duplicates, and showed as mean ± SEM. Significance was calculated by Student’s* t-test* (bar graphs) or *one-way Anova* (disease score), ns: non-significant.

To determine whether LRV1-mediated upregulation of NLRC2 or NLRC5 in macrophages plays a pathogenic role in *LgyLRV1+* infected mice, we investigated disease progression among mice deficient in NLRC2 or NLRC5 as compared to WT mice. Mice lacking NLRC2, showed significantly reduced parasite load and lesion swelling (Fig 7A and 7B). Oppositely, no discernible difference was seen between WT and NLRC5^-/-^ mice (Fig 7C and 7D). These results indicate that LRV1 promotes the virulence of its microbial host partially through NLRC2 in mice.

## DISCUSSION

The innate immune response to *Leishmania* formed within the first hours after parasite inoculation is a determining factor for the outcome of leishmaniasis 1. We previously revealed that the dsRNA genome of an endosymbiotic virus within virulent *Leishmania* species is able to act as a potent innate immunogen that worsens the outcome of cutaneous disease 5. Although we showed the essential role of TLR3, we could not formally exclude the role of other PRRs in leishmaniasis such as the RIG-like receptors (RLRs) and various members of the NOD-like receptors (NLRs).

In this study, we found that macrophage infection with *LgyLRV1*+ parasites resulted in the transcriptional up-regulation of several inflammasome components such as caspase-1, IL-1β, NLRP3, and AIM2. These results mirrored those of a preliminary effort to profile the transcription of inflammasome components in a macrophage system of infection 25. However, we found that *L. guyanensis* infection did not activate the inflammasome, irrespective of its LRV1 status. Notably, an infection in primed macrophages was unable to activate caspase-1 or IL-1β and mice deficient in the key inflammasome components ASC and caspase-1/11 showed no change in their disease phenotype. Further, we found no role for RLR-signalling in response to LRV1, as removing its key signalling adaptor protein MAVS had no effect on cytokine production or parasite burden *in vitr*o, nor did this defect have any impact on disease progression *in vivo*. As MAVS is also a central or synergising adaptor protein for other non-RLR pathways, the unchanged phenotype in MAVS^-/-^ mice also excluded the RLR-independent pathway of virus-induced apoptosis via MAVS-MKK7-JNK2 47. The absence of a role for RLR signalling indicated that LRV1 was likely not able to exit the phagolysosome to engage these receptors in the cytoplasm and that LRV1 dsRNA recognition was limited to endosomal TLR3.

NLRs are able to detect both PAMPs and DAMPs, which are present in the destructive inflammatory environment of a typical leishmaniasis lesion. NLRs have so far proven protective in murine leishmaniasis caused by *L. amazonensis*, *L. braziliensis *and *L. infantum*. This protection was elicited by an NLRP3-dependent production of nitric oxide (NO) involving IL-1β 24. Upon incubation with LPS, the resultant active IL-1β induced by NLRP3 signalling carried out this response in an autocrine manner, whereby the IL-1 receptor and its MyD88 adaptor protein were necessary and sufficient to trigger parasitotoxic oxidative stress via inducible nitric oxide synthase (iNOS). It should also be noted, however, that LPS can elicit a non-canonical activation of the inflammasome in the cytosol via caspase 11 and its CARD domain 48. It is likely that production of IL-1β is not sufficient to control the disease for every *Leishmania* species, since *L. major* was shown to induce IL-1β in BMDMs when exposed to LPS, but lesion sizes were not increased in caspase-1 deficient mice 24. Another group showed that blockade of IL-1β signalling rendered BALB/c mice resistant to the infection 27. Oppositely, NLRP3 inflammasome activation was required to establish non-healing infection with a particular strain of *L. major* 26. Our data showed that, even in the presence of LPS, *Lgy* parasites did not activate the inflammasome. Therefore, the question of species-specific inflammasome activation and evasion remains to be determined. Of note, *Leishmania* parasites can be abundantly coated with the leucine-rich-repeat (LRR)-enriched surface antigen PSA-2. Interestingly these antigens vary across *Leishmania* species, and have been associated to increased virulence 49, which could explain the discrepancies in inflammasome activation between different *Leishmania* species and/or strains. Aside from parasite species, we cannot yet exclude that other factors such as the site of infection (footpad or ear) and the dose of parasites injected, which differ remarkably between reports, could participate in inflammasome activation.

Inflammasome evasion could be an essential virulence strategy in cutaneous leishmaniasis. This evasion could occur via the catalytic activity of GP63, a ubiquitous metalloprotease on the surface of the* Leishmania* parasite, which can cleave NLRP3 and inflammasome components as shown *in vitro *but no evidence on the impact of GP63 has been provided to date *in vivo* 50. Alternatively, the deubiquitinating enzyme A20, which is a negative regulator of NF-κB and TLR signalling in *L. donovani* infection 32 was attributed a new role in suppressing inflammasome activity 31,33,51. We found that, similarly to *L. donovani*, *L. guyanensis* infection induced A20 expression in macrophages. Interestingly, at 4 hours post infection, A20 expression was higher in *LgyLRV1+* infected macrophages, compared to *LgyLRV1- *or Poly (I:C) stimulation, suggesting an additive role for TLR3 stimulation. It will be interesting to assess whether the same inhibition of inflammasome activation by A20 is observed in models of Leishmania/virus co-infection where exogenous viral infection exacerbates the severity of *L. guyanensis* infection, promoting parasite dissemination and disease relapses, reproducing the phenotype of *LgyLRV1+* infection 52.

The formation of an inflammasome is only one of the many possible signalling pathways of NLRs. Many NLRs respond to viral cues in inflammasome independent pathways. We found that *LgyLRV1+* infection, or poly(I:C) treated macrophages, up-regulate the inflammasome independent NLRs: NLRC2 and NLRC5. Moreover, we showed that the infection of mice deficient in NLRC2 but not NLRC5 led to a reduction in parasite burden and lesion size. These results indicated that there could be an NLRC2-mediated host dependent virulence mechanism other than the LRV1-mediated inflammatory response. NLRC2 drives the recruitment of monocytes to the site of infection 42. The LRV1-mediated hyperinflammatory response that promotes parasite survival could require NLRC2-dependent monocyte infiltration, cells which could in turn serve as secondary host cells, thus explaining the reduction of disease severity in NLRC2^-/-^ mice. This hypothesis will require further investigation.

NLRC5 (CITA) is responsible for MHC-I transcription. The role of MHC-I on CD8 cells in leishmaniasis shows a distinct disease type-specificity as CD8+ T cells are detrimental to mucosal and metastatic infections 53, but protective in simple cutaneous leishmaniasis. The pathogenic role of CD8+ T cells in *L. braziliensis* infection was shown to be due to tissue-destructive levels of granzyme B 54. It is thus interesting that NLRC5 deficiency in mice had no effect on disease phenotype in *L. guyanensis* infection irrespective of LRV1 content, indicating that CD8+ T cells possibly do not play an important role in LRV1-mediated pathology.

Taken together, we were able to show that *L. guyanensis* evades inflammasome activation, where no central components of the inflammasome impacted pathology in a murine model of disease, irrespective of the presence of LRV1. Additionally, we excluded the involvement of RLR receptors in the development of *Lgy* pathology, and confirmed that LRV1 does not stimulate this cytoplasmic dsRNA receptor. Considering the potently protective role of inflammasome activation previously found in infections by other *Leishmania* species, these results indicated that inflammasome evasion might be an important mechanism of virulence in *Leishmania* infection. Finally, as NLR and RLR pathways are the major non-TLR3 routes of dsRNA-induced inflammation, this work confirmed TLR3 as the major pathway of inflammatory pathology in LRV1-mediated cutaneous leishmaniasis.

## MATERIALS AND METHODS

### Ethical Statement

All animal protocols in this publication were approved by the Swiss Federal Veterinary Office (SFVO), under the authorization numbers VD2113.1 and VD2113.2. Animal handling and experimental procedures were undertaken with strict adherence to ethical guidelines set out by the SFVO and under inspection by the Department of Security and Environment of the State of Vaud, Switzerland.

### *In vitro* parasite culture

Two isogenic clones of *L. guyanensis* (MHOM/BR/75/M4147) showing uniformly high levels of LRV1 or completely lacking it were described previously 55. We refer to these clones as *LgyLRV1+* and *LgyLRV1-*, respectively. Both strains express a firefly luciferase gene (LUC), integrated stably into the locus of the small ribosomal subunit and displayed comparable luminescent efficacy 55. In addition, two strains of *L. infantum *were used. The first one was isolated from a dog in Spain (JPC MCAN/ES/98/LLM-722) and will be referred as *Linf *S1, while the second one was isolated from a human patient in Switzerland (MHOM/CH/2016/BELA) and will be referred as *Linf *S2. Parasites were cultured *in vitro* as promastigotes at 26°C in freshly prepared Schneider’s insect medium (Sigma) supplemented with 10% heat-inactivated fetal bovine serum (PAA®), 10mM HEPES and 50U/ml penicillin/streptomycin (Animed®), 0.6mg/L biopterin and 5mg/L hemin (Sigma-Aldrich®). Each passage yielded infectious metacyclic promastigotes after 6 days and stocks were kept no longer than 5 passages.

### Murine model of leishmaniasis

All mice were bred in a specific pathogen-free housing facility at the University of Lausanne, Switzerland and backcrossed at least 10 times onto a C57BL/6 genetic background (purchased from Harlan Laboratories, Netherlands). Caspase-1/11^-/-^ and NLRC2^-/-^ were purchased from Jackson Laboratories (B6.129S2-Casp1tm1Flv/J and B6.129S1-Nod2tm1Flv/J respectively). The following genetic knock-outs were a kind gift from Jürg Tschopp and previously described: MAVS^-/-^, ASC^-/-^, IL-1β^-/-^, and NLRC5-/- 36,56,57,58. Age-matched (6-8 weeks old) female mice were infected in the hind footpads with 3x10^6^ stationary phase *LgyLRV1+ *or *LgyLRV1- *promastigotes. Change in footpad swelling was measured weekly using a Vernier caliper as a proxy for disease score.

### Parasite quantification by luminescence

At the peak of infection (week 4/5) parasite burden was quantified using *in vivo* imaging able to detect luminescence emitted by luciferase-transfected parasites. Briefly, mice were injected intra-peritoneally with D-Luciferin sodium salt (Regis technologies®) prepared in PBS at a final concentration of 15 mg/kg. After a 10min incubation, mice were anesthetized by continuous gas anesthesia and imaged using a Xenogen Lumina II imaging system (IVIS®, 10min exposure time). Total photon flux over the leishmanial lesions in the footpads was assessed using the associated software (Living Image®).

### BMDM culture, infection and stimulation

Bone marrow cells were extracted from naive C57BL/6 mice. Erythrocytes were removed by lysis using ACK lysis buffer (1,5M NH_4_Cl, 100mM KHCO_3_, 10mM EDTA-2Na) and then incubated with macrophage differentiation medium (complete DMEM supplemented with L929-conditioned media) at 37°C. After 6 days of differentiation, adherent BMDMs were isolated and plated at 1x10^6^/ml in 48 well plates before being stimulated. Inflammasome activity assays used a pre-stimulation step with 200 ng/ml LPS (Sigma-Aldrich®) for 3 hours. Cells were then washed and infected (1:10) with stationary phase *LgyLRV1+ *or* LgyLRV1- *parasites or stimulated with the synthetic TLR3 ligand, poly(I:C) (2 µg/ml, Sigma-Aldrich®) for 24 hours at 37°C. A positive control was treated with 5 µM nigericin (Sigma-Aldrich®) for 1 hour, while a negative control was left unstimulated after LPS pre-treatment. Alternatively, for A20 measurement, BMDMs were infected (1:10) with stationary phase* LgyLRV1+*,* LgyLRV1-*, *Linf *S1 or *Linf* S2 parasites or stimulated with poly(I:C) for 4 or 8 hours at 37°C.

### Western blot

BMDMs were lysed with a mixture of RIPA Buffer IV (Biotech RB4448), and a Protease/Phosphatase inhibitor cocktail (Cell Signaling 5872S) in PBS. Laemmli’s Sample Buffer was added before lysates were size-fractionated by SDS-PAGE and wet-transferred to a nitrocellulose membrane. Western Blotting was performed using the following antibodies: rabbit polyclonal anti-mouse caspase-1 p10 (Santa Cruz, SC514), goat polyclonal anti-mouse IL-1β (R&D Systems, AF-401-NA), rabbit anti-mouse A20 (Cell Signaling, D13H3), mouse monoclonal anti-mouse γ-tubulin (Sigma-Aldrich® T6557), goat anti-rabbit IgG HRP conjugated (Promega W4011) and goat anti-mouse IgG HRP conjugated (Promega W4021). Where indicated, protein expression was quantified with densitometric analysis using ImageJ software.

### High Content Microscopy

BMDMs were counted with Vi-Cell® (Beckman Coulter). Then, 0.1x10^6^ BMMs were seeded on Falcon® 96 Well clear-bottom Tissue-Culture treated plate. Cells were infected with stationary phase *LgyLRV1+* or *LgyLRV1-* parasites (1:10) for 24 hours and then fixed with freshly made 3.7% PFA in PBS. Cells were subsequently stained with DAPI to visualize the nuclei of parasites and macrophages and washed in PBS using a Biotek MultioFlo FX plate washer. 49 (7x7 square) images from each well with x40 lens were acquired using ImageXpress Micro XLS and analyzed using MetaExpress software.

### Statistical analysis

Statistical significance was determined using an unpaired parametric *t-test* (for single-point analysis on bar-graphs) or a *one-way Anova* (x/y disease curves) as calculated by GraphPad® software (Prism™ v5a). Significance was recognized when p <=0.05 and represented in three ranks namely *: p <= 0.05, **: p <=0.01 and ***: p <= 0.001.

## SUPPLEMENTAL MATERIAL

Click here for supplemental data file.

All supplemental data for this article are also available online at http://microbialcell.com/researcharticles/leishmania-guyanensis-parasites-block-the-activation-of-the-inflammasome-by-inhibiting-maturation-of-il-1β/.
