# Supplementary material for: Leishmania guyanensis parasites block the activation of the inflammasome by inhibiting maturation of IL-1β
Source: Microb Cell. 2018 Jan 14;5(3):137–49. doi: 10.15698/mic2018.03.619 (PMC5826701; doi:10.15698/mic2018.03.619)
Supplement: Supplementary file 1 [file mic-05-137-s01.pdf]

Supporting Information

Fig S1. The deficiency of ASC in Itgax (CD11c), Lysosome 2 (Lysm) or Keratin 14 (K14) expressing cells does not affect LRV-mediated pathogenesis in mice. Mice were infected with $3x10^6$ stationary promastigotes of *LgyLRV1+*. Footpads of mice were measured weekly. All conditional knockout mice were homozygous for floxed ASC alleles and expressed Cre on one allele under the control of indicated promoters. Non-cre expressing littermate mice were used as a control.

Supplementary Figures

Figure S1

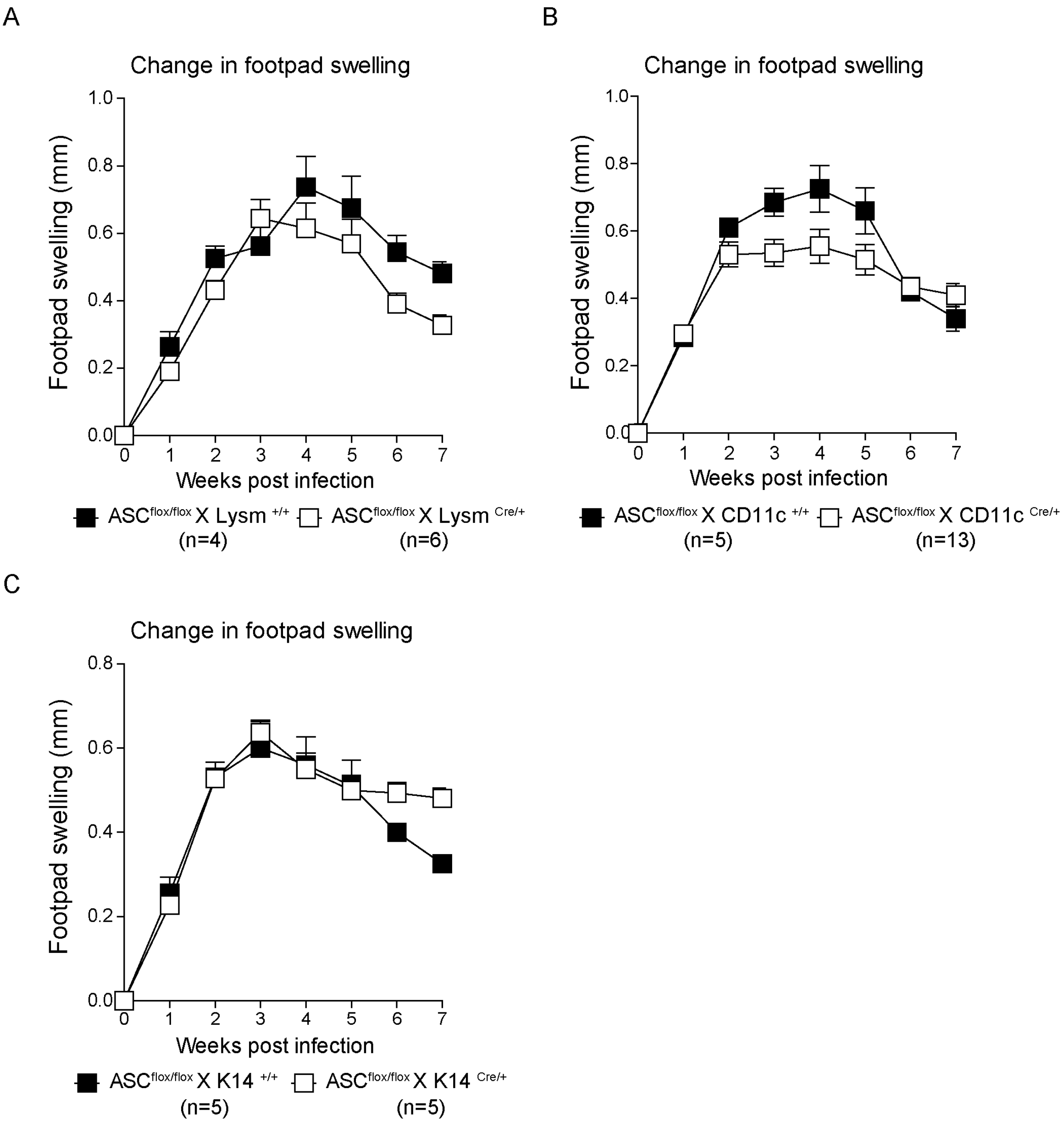

Table S1. The list of primers used for qRT-PCR.

Gene	Forward Primer	Reverse Primer
Aim2	GTC ACC AGT TCC TCA GTT GTG	CAC CTC CAT TGT CCC TGT TTT AT
Asc	GAG CAG CTG AAA CGA CTA A	GCT GGT CCA CCA AGT GTC CT
Casp1	ACA AGG CAC GGG ACC TAT G	TCC CAG TCA GTC CTG GAA ATG
Il1b	CAA CAA ACA AGT GAT ATT CTC CAT	GTG CCG TCT TTC ATT ACA CAG
L32	AAG CGA AAC TGG CGG AAA C	TAA CCG ATG TTG GGC ATC AG
Naip5	TGC CAA ACC TAC AAG AGC TGA	CAA GCG TTT AGA CTG GGG ATG
Nlrc2	CCA ATC TTC ACG TCG TCT CA	ACG TGC TGT AGA AGG AAG GC
Nlrc3	CCT CAG CCT GCG AGA AAA CT	TCG GTC ATG GAG GAG ATT AGC
Nlrc4	TCC CCT CCA ACT GCT TCA AC	GAA AAG GAT GGG AAT GAA GCT C
Nlrc5	TGG AGG AGG TCA GTT TGC	ATG CTC CTG ATT GCT GTG TAG
Nlrp1	ATG TGG ACC CAA CCT TCA AA	GTA CGT GCT CCT GGA AAG GT
Nlrp3	AAG TAA GGC CGG AAT TCA CC	GCT CCA ACC ATT CTC TGA CC
Nlrp10	TGC TCC GTA CAT TGA AAT CAG TT	TCA AGA CGC TGA AGT TCC ACT
Nlrp12	GAGCGA GGA CAG GGA GAA GA	TTG CGG TCT TCC ATT AGC TG
Nlrx1	CAT GAA AAC TCG GCA GAC AG	GGC TAA ACC ACT CGG TGA GG
